# Researches on the Possibility of Temporarily Restoring to Life Individuals Dying of Disease

**Published:** 1859-07

**Authors:** E. Brown-Sèquard


### ECLECTIC DEPARTMENT,

### AND SPIRIT OF THE MEDICAL PERIODICAL PRESS.

---

*Researches on the Possibility of temporarily Restoring to Life Individuals Dying of disease.* By Dr. E. Brown-Séquard. Translated from the *Journal de la Physiologie de l'Homme et des Animaux*, by George S. Blackie, M. D.

For a number of years my researches on the Transfusion of Blood, commenced in 1846 and continued up to the present time without interruption, have often given results which show positively that life may be restored for a certain time in mammalians dying of some diseases, more particularly of peritonitis.

I shall report here some of the principal facts I have observed; as I intend to publish the rest at a future time, in a memoir on the Transfusion of the Blood, which I am preparing.

The experiments which have been successful, were performed on animals which had not breathed for a variable number of minutes (17 in one case), and in whom every trace of voluntary motion and sensation had disappeared. In most of the cases the sounds of the heart could be heard; but no pulse could be distinguished in the principal arteries of the limbs, not even, often, in the carotids; in two cases the beating of the heart appeared to have entirely ceased for several minutes. The last convulsive throes of death had occurred, the pupil was fast dilating, or had already dilated. In a word, death was imminent; and certainly no one could think that under such circumstances a spontaneous return to life was possible.

The diseases which had brought the animals to this state of imminent death that I am about to describe, were for the most part inflammations of the peritoneum or pleura, following wounds of these membranes made during experiments on the different abdominal viscera or on the vagi, sympathetic or diaphragmatic nerves in the thoracic cavity. In some cases the approaching death was due to the extirpation of the supra-renal capsules, or of the kidneys.

In every case, without exception, the proximate cause of death was asphyxia. I do not wish to examine here how that asphyxia was produced; it is a question of too much importance for mere incidental consideration. I intend to make this the object of a series of papers on the cause of death in the acute diseases of man and animals. But I ought to say at present that in a very large number of the cases of the lesions of the abdominal viscera, in the experiments on which the present paper is founded, the asphyxia was for the most part the result of an enfeebling of the movements of the heart, an enfeebling, of which the *principal*, but not the only cause, was the irritation of the branches of the great sympathetic nerve within the abdomen.

But to whatever cause we attribute the production of the asphyxia, there it was; and in attempting to restore life for a certain time to dying animals, it had to be considered. I was thus led to try if artificial inspiration might not have some influence, and I employed it in eight or ten dying animals. In a few of these, there was a limited increase of strength in the force of the movements of the heart, and one animal only seemed to return to consciousness for a few minutes. It is quite different when the artificial respiration is employed before the death agony; but that is not the subject of this paper. I will limit myself to saying that we may generally stave off the death struggle from one to several hours, by means of pulmonary insufflation.

That insufflation being incapable of restoring to life animals dying of febrile diseases, I have tried to find if I could not succeed better by employing other means. To those who imagine that galvanism may be very useful as a means to re-establish life in asphyxiated animals, I wish to say that I did not employ it a single time; because I knew that the best way to destroy the remnant ot life in dying animals is, to submit their nerves and muscles to the exhausting excitement caused by galvanism.

I thought then of employing transfusion of blood; not with the insane idea of the early transfusors, who believed that they could cure the most serious ailments by substituting healthy for diseased blood; but desiring to see what influence that operation would exert on an organism scarcely living, and necessarily condemned to a speedy death.

I performed the operation sometimes in one way, sometimes in another; but in the space afforded to me here, I shall confine myself to reporting one experiment which I performed, and afterwards I shall give a general summary of the various modifications of the experiment.

*Experiment.*—In October, 1851, a dog from which I had cut out the great abdominal sympathetic nerve, was attacked with peritonitis, and after being two or three days sick, presented those signs of speedy dissolution so well known to vivisectors. Voluntary motion had ceased some time (I did not note exactly how long); sensation and reflex action had ceased altogether, even in the eyes. The last respiratory movements (those of the jaws and nostrils) had ceased. The death struggle (very weak in this case) of the limbs, the face and the eyes, consisted merely of a slight tremor, limited to a very few muscles. The animal expelled its feces and urine; the pupils were dilated and the movements of the heart could not be felt. The only sign of life which remained consisted in an indefinite sound, not at all like the sound of the heart, which was heard eight times in the twelve seconds which immediately preceded the transfusion of the blood of another dog, performed in the following manner: A tube of silver, having the form of the letter T, was fixed by the branch which corresponds to the top of the T, in the interior of the right carotid of the dying dog; and the free extremity, somewhat curved, of the other branch, was introduced and fixed into the left carotid of the second dog, whose head and limbs were firmly secured to the table. Immediately the arterial blood of the healthy dog circulated in two opposite directions, towards the head and toward the heart in the carotid of the dying animal. At the same time the left jugular vein and one of the femoral veins of the dying dog were opened. These two veins at first did not bleed; but almost immediately the jugular did, and after twenty or thirty seconds the femoral commenced to yield a little. During the first fifteen seconds, by pressing the carotid which furnished the transfused blood, I diminished the quantity of that liquid, and in two minutes the operation was complete, and ligatures were placed on the carotid and vein of the dying animal. The jugular vein was left open four or five minutes, during which the heart's movements began to be perceived. In proportion as the blood flowed from the jugular vein, the pulse returned. I then had recourse to artificial respiration. As I had only one assistant, the disorder caused by the experiment, prevented my counting the pulse during the first twelve minutes. When I could at last count it, it was very feeble, and had sixty-four strokes in the minute. Insufflation was continued almost without interruption for half an hour. During the eighth minute after its commencement there was sensibility of the cornea; a few minutes more, and inspiratory movements were seen. At last, after twenty minutes, the animal performed voluntary movements; and when artificial inspiration was stopped, respiration occurred with frequency, but not with strength. The restoration to life, however, was complete as far as the existence of the principal functions of the animal and organic life are concerned. The animal, although feeble, got upon his legs, and wagged his tail when caressed. The pulse, though small and feeble, was rapid (110 to 120, some hours later, 80). Finally, after remaining in this state four or five hours, the animal sunk again and died (I had almost written *died again*), eleven hours and a half after the transfusion.

I need add but a few more remarks to the details of this experiment. I had not weighed the dogs on which I experimented, and I do not know what quantity of blood was transfused, nor how much was lost by the dying dog. But I observed that the animal furnishing the transfused blood was small, and the other large; so considering these facts and the duration of the transfusion, and several other circumstances, I have cause to believe that the dying dog did not receive more blood than he lost. But I have learned from subsequent experiments, the quantity of blood he received was too large, and it is likely that he would have been more speedily resuscitated had that quantity been diminished.

There were in this experiment several causes for the return to life:— 1, the passage of the normal arterial blood through the coronary arteries, giving more irritability to the muscular fibres of the heart, and consequently rendering them more capable of obeying the stimuli (whatever they are), which cause them to counteract rhythmically; 2, the passage of normal arterial blood through the encephalic arteries; 3, the partial substitution of normal blood for blood altered by an inflammatory disease, and by the asphyxia produced in the death struggles; 4, the pulmonary insufflation; 5, the bleeding from the jugular vein, thus relieving the engorgement of the heart.

Of these five causes of the temporary return to life, there is one—insufflation—which, I have already said, employed alone, is incapable of producing this result. With regard to the others, I can say that employed alone they are almost as insufficient as insufflation. For instance, the injection of arterial blood to the heart by the carotid in dying animals, is capable merely of increasing for some time the force and rapidity of the movements of that organ, but not to restore the circulation; also, the transfusion of blood by a vein can only hasten the complete cessation of the movements of the heart, if it is done without the right ventricle being enabled by an opening in the jugular vein to disgorge itself of the blood which prevents its contraction in asphyxia. Besides, the transfusion of the carotid towards the brain only adds to the difficulties of the contractions of the right ventricle; and lastly, the opening of the jugular vein alone, makes the heart more capable of acting freely, but only for a short time.

A long time ago, M. Segalas,* and after him John Reid,† Dr. Cormach,‡ Dr. H. Londsale,§ and finally Dr. Struthers,‖ have demonstrated that in asphyxia from hanging, from blows on the head, and from certain poisons, the movements of the heart could be rendered more active, or could be restored if they had ceased, by bleeding at the jugular, and by this means relieving the right cavities of the heart of their engorgement. This excellent method employed alone on an animal dying in consequence of inflammation, is absolutely inefficacious. Even when using along with it forced respiration, I have never succeeded in re-establishing the circulation, and still less the respiration and the functions of animal life. The only result of combining the two means, has been an acceleration of the movements of the heart. But when I have employed both these at the very beginning of the death struggle, or, better, before the convulsions began, I have often seen the respiration and circulation restored one or two hours, and sometimes there was a momentary return to the functions of animal life.

I tried, in a number of cases, transfusion by the carotid in two opposite directions, without employing insufflation, and taking care to keep the jugular vein open during and a little after the transfusion. The return to life in a case of this sort was rarer than when insufflation was employed, but it did occur in several animals, who survived two or three hours.

I cannot tell what is the exact proportion, among the number of animals on whom I operated, of those who were restored temporarily to life; nor

---

* *Journal de Physiologie, de Magendie*, vol. iv., 1824, p. 290.
† Physiological, Anatomical and Pathological Researches. 1848, p. 50–60.
‡ Treatise on Creasote. 1839, p. 84.
§ Edinburgh Medical and Surgical Journal, 1836, No. 138.
‖ The same Journal, 1856, p. 241, 56.

about what was the average length of this new life imparted to the dying, on whom I practiced transfusion, insufflation, and bleeding from the jugular; because I have not all the records of my experiments at hand; but from notes which are at present before me, I learn that in eleven experiments made on adult dogs, cats, and rabbits, nearly all dying of peritonitis, four of the subjects were entirely restored to life for two, three, and four hours (one dog, one cat, and two rabbits); that three others recovered their circulation, respiration, and reflex action for one or two hours, without, however, voluntary motion or sensation; and that in the other four there was no result but a slight acceleration of the heart's action.

After numerous experiments on the transfusion of blood, carried on in the last few years, having learned that the transfused blood does not need either to be warm or to contain fibrin,* I have ceased to employ a healthy animal from which to obtain transfused blood. After having bled at the jugular and commenced insufflation, I injected at several intervals, and very slowly, a quantity of blood somewhat less than the animal had lost. I injected alternately towards the heart and the head, in order to act on the brain for the purpose of re-establishing the respiration, and on the muscular fibres of the heart to increase their irritability.

From these experiments can we arrive at any conclusions relative to the combined employment of transfusion, insufflation, and bleeding from the jugular in men dying of inflammatory or other diseases? It is evident that in an immense majority of cases it would be useless, if not cruel, to snatch from death, for a time necessarily very short, an individual of our species condemned to death by material and irreparable lesions. But cases present themselves in which it would be of immense importance, could intelligence, speech, the senses, and voluntary motion be restored to the dying for a few minutes only. Now the fact mentioned in this article, showing that *all the functions of animal life can be re-established for some hours in animals in whom the death struggles have almost entirely given place to death*, render it extremely probable that the intellectual faculties, the senses, speech, etc., could be re-established for some hours in individuals who had lost their faculties, and in whom the death struggles had already commenced.

The success of these various operations is the more probable, because in performing them we need not wait, as I did in the case of animals, until the death struggles had made considerable progress, and still less, until they have entirely ceased.

---

* I have recently found, however, that defibrinated blood presents this danger, that when mingled with the blood of the animal into which it is transfused, it sometimes excites a sudden coagulation; but it is probable that by adding 1-1000th or 1-1500th part of caustic ammonia to the defibrinated blood, we can avoid the only danger I know of in its use.

